# 7,8-Dihydroxyflavone Enhances Cue-Conditioned Alcohol Reinstatement in Rats

**DOI:** 10.3390/brainsci10050270

**Published:** 2020-05-01

**Authors:** Samuel J. Hogarth, Elvan Djouma, Maarten van den Buuse

**Affiliations:** 1 School of Psychology and Public Health, Department of Psychology and Counselling, La Trobe University, Melbourne 3086, VIC, Australia; s.hogarth@latrobe.edu.au; 2 School of Life Sciences, Department of Physiology, Anatomy and Microbiology, La Trobe University, Melbourne 3086, VIC, Australia; e.djouma@latrobe.edu.au; 3 Department of Pharmacology, University of Melbourne, Melbourne 3010, VIC, Australia; 4 The College of Public Health, Medicinal and Veterinary Sciences, James Cook University Townsville, Townsville 4811, QLD, Australia

**Keywords:** brain-derived neurotrophic factor (BDNF), alcohol use disorder, reinstatement, relapse, operant self-administration

## Abstract

Alcohol use disorder (AUD) is a detrimental disease that develops through chronic ethanol exposure. Reduced brain-derived neurotrophic factor (BDNF) expression has been associated with AUD and alcohol addiction, however the effects of activation of BDNF signalling in the brain on voluntary alcohol intake reinstatement and relapse are unknown. We therefore trained male and female Sprague Dawley rats in operant chambers to self-administer a 10% ethanol solution. Following baseline acquisition and progressive ratio (PR) analysis, rats were split into drug and vehicle groups during alcohol lever extinction. The animals received two weeks of daily IP injection of either the BDNF receptor, TrkB, agonist, 7,8-dihydroxyflavone (7,8-DHF), or vehicle. During acquisition of alcohol self-administration, males had significantly higher absolute numbers of alcohol-paired lever presses and a higher PR breakpoint. However, after adjusting for body weight, the amount of ethanol was not different between the sexes and the PR breakpoint was higher in females than males. Following extinction, alcohol-primed reinstatement in male rats was not altered by pretreatment with 7,8-DHF when adjusted for body weight. In contrast, in female rats, the weight-adjusted potential amount of ethanol, but not absolute numbers of active lever presses, was significantly enhanced by 7,8-DHF treatment during reinstatement. Analysis of spontaneous locomotor activity in automated photocell cages suggested that the effect of 7,8-DHF was not associated with hyperactivity. These results suggest that stimulation of the TrkB receptor may contribute to reward craving and relapse in AUD, particularly in females.

## 1. Introduction

Alcohol Use Disorder (AUD) can be defined as the compulsive and escalating consumption of alcohol in spite of severe detrimental consequences. AUD affects approximately 240 million people, or 4.9% of the global population [1], and has a significant impact on socioeconomic frameworks within society. Poor societal outcomes in AUD manifest as a result of social dysfunction, reduced workplace productivity, and physical illness that typically results in high healthcare expenses [2]. The exact mechanisms underlying AUD remain largely unknown and current intervention strategies are limited [3]. Consequently, understanding the mechanisms which contribute to neurochemical adaptations to excessive alcohol consumption is vital in the development of novel therapeutic treatment options.

The mesolimbic and mesocortical neuronal pathways projecting from the ventral tegmental area to the nucleus accumbens and prefrontal cortex have been identified as core components of addiction development [4]. These neurocircuitry systems are regulated by neurotrophins, which are responsible for neural maintenance, development and plasticity, and their expression is altered in response to the abuse of drugs such as alcohol [5]. Of the four neurotrophins expressed within the mammalian central nervous system (CNS), brain-derived neurotrophic factor (BDNF) is the most abundant and widespread and has been implicated in drug-induced neuroadaptations in AUD and the regulation of regions of the brain crucial for addiction development [6]. 

BDNF is generated as the precursor, proBDNF, where it is cleaved to the mature form (mBDNF) [7]. Upon release, mature BDNF binds to the tropomyosin receptor kinase B (TrkB) to initiate downstream signalling pathways which regulate gene expression encoding proteins involved in neuronal cell survival, axon and dendrite growth, and plasticity [7]. Alcohol intake alters BDNF expression in rats and mice over acute and chronic ingestion [5]. For example, moderate ethanol consumption in mice has been shown to promote BDNF expression within the dorsal striatum [8], and prolonged exposure to alcohol results in a reduction in cortical levels in the brain [9]. Furthermore, a reduction in BDNF within the nucleus accumbens (NAc) has been detected in alcohol-preferring rats [10], and lower levels of the neurotrophin in the medial prefrontal cortex (mPFC) are correlated with escalated alcohol consumption [11]. The BDNF val66met polymorphism, which results in reduced activity-dependent BDNF release in the brain [7,12], is associated with enhanced alcohol intake in mice [13]. We recently used an operant alcohol self-administration paradigm to test the reinstatement/relapse propensity of BDNF heterozygous (BDNF HET) rats, which have a 50% endogenous reduction in BDNF levels in the brain [14]. We found that female, but not male, BDNF HETs displayed enhanced reinstatement potential compared to their sex-matched wildtype counterparts. These findings suggested that BDNF is a negative regulator of alcohol intake and AUD and this role may be sex-dependent.

While changes in BDNF signalling have been suggested as potential mediators of alcohol use, few studies have attempted to induce alterations in neurotrophic signalling and assess the effect on alcohol intake. Such studies might inform on the potential efficacy of treatments aimed at BDNF signalling in AUD. In a previous study in rats, BDNF infusion into the dorsolateral striatum gated alcohol intake and terminated drinking episodes [15]. In mice, the TrkB receptor agonist, LM22A-4, attenuated enhanced alcohol consumption [12]. From these findings, it can be hypothesized that the activation of TrkB, either with BDNF or a TrkB agonist, could be a potential therapeutic lead for the prevention of AUD. 7,8-Dihydroxyflavone (7,8-DHF) is a selective and potent small-molecule agonist of TrkB that can permeate the blood brain barrier (BBB) and is orally bioavailable [16]. This potent TrkB agonist has recently been implemented in a range of behavioural intervention models including studies in spatial memory [17], intracerebral haemorrhage [18], obesity [18], Parkinson’s disease and Alzheimer’s disease [19]. However, there has been no previous investigation into the effects of 7,8-DHF on alcohol use and addiction pathophysiology, particularly relapse, where we found an involvement of BDNF in our previous studies [14]. Following this previous study in BDNF HET rats, we therefore examined the effect of subchronic 7,8-DHF administration on reinstatement/relapse in an operant alcohol self-administration paradigm in male and female rats. 

## 2. Materials and Methods

### 2.1. Animals

Male and female wildtype Sprague Dawley (SD) rats were obtained from the Animal Resource Centre (ARC; Murdoch, WA, Australia) at 5 weeks of age and housed at the La Trobe Research and Teaching Facility, Melbourne, for the duration of the study. Forty-eight rats were kept in sex-matched groups of four in individually ventilated cages (Tecniplast, Italy) and allowed to habituate for 1 week prior to the commencement of behavioural testing. 

### 2.2. Operant Ethanol Self-Administration

**Figure 1:**
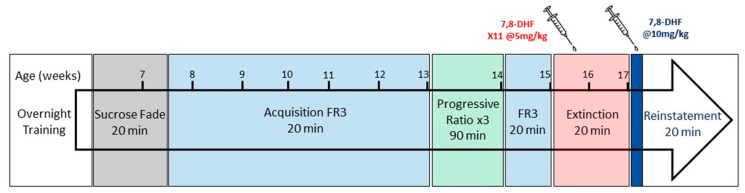
Experimental timeline of treatment protocol depicting the stages of operant training and response. Rats were trained in 1 overnight session followed by a sucrose fade, and lever presses were recorded during acquisition, progressive ratio, extinction, and reinstatement. FR = Fixed Ratio.

Twenty-four male and 24 female rats were trained in operant chambers (Med Associates, St Albans City, VT, USA) as previously described [20]. Each chamber contained two levers at opposite corners of the arena. A cue light was positioned above each lever and indicated when the sufficient number of presses was reached to obtain a reward, resulting in either 100 microlitres of ethanol solution or water being dispensed into a fluid receptacle adjacent to each lever. Vanilla essence was placed under the active lever as an olfactory cue to indicate the side of the cubicle containing ethanol solution. Rats were first habituated to the operant chambers over a 16-h overnight session and allowed to press the levers for a reward containing sucrose and a 5% *v*/*v* ethanol solution (two presses per reward). Food pellets were placed in the bottom of the chamber to prevent any food restriction. Following overnight training, rats were habituated to the aversive taste of alcohol in daily 20-min sessions over 2 weeks using a previously described sucrose fade protocol [14]. Upon complete withdrawal of the sucrose solution, the active lever dispensed a 10% ethanol solution for the remainder of the experiment (Figure 1).

### 2.3. Acquisition

Following successful completion of the sucrose fade, rats were trained daily (Monday–Friday) in 20-min operant sessions, at a fixed-ratio response of three (FR3, three presses equals one reward). During this acquisition phase, the number of lever presses recorded per session increased over time until the majority of rats reached a stable baseline within 25 days of acquisition training (Figure 1). Similar to overnight training and sucrose fade protocols, a drop of vanilla essence was placed under the active lever as an olfactory cue to indicate the ethanol-containing receptacle. 

### 2.4. Progressive Ratio

To ascertain that the same protocol was used as in our previous study [14], once lever pressing had stabilized, 90-min progressive ratio (PR) sessions were included to measure the breakpoint of responding to the active lever (Figure 1). This was conducted every second day over a 1-week period in which the number of lever presses required to obtain a reward was incrementally increased with every subsequent reward. For example, the first reward occurred after one press, the second reward after two additional presses and the third after three additional presses. The water lever followed the same pattern of accumulative presses. The three PR sessions that were conducted were subsequently averaged to give a mean value per animal for breakpoint analysis. The breakpoint of each rat represented the number of total presses or g ethanol per kg body weight that the animal reached before giving up on obtaining a reward within a 90-min period [21]. 

### 2.5. Extinction and Reinstatement

After completing PR, rats were re-baselined over a 1-week FR3 period before commencing the extinction phase (Figure 1). Extinction training began on experiment day 35 and involved the removal of both the ethanol reward and water solutions from the operant chamber. The stimulus light and olfactory vanilla essence cues were also removed, and rats were allowed to lever press for 20-min sessions over 2 weeks until the number of active presses became low or equal to the nonactive lever. On the 5th day of extinction, daily intraperitoneal (IP) injections of 5 mg/kg 7,8-DHF (TCI Chemicals, Tokyo, Japan) in 10% DMSO/saline were introduced to half of the male and female rats directly following the operant session. The remaining rats received a vehicle IP injection with the same 10% DMSO/saline solution. The treatment regimen of 7,8-DHF was chosen on the basis of previous studies, showing that subchronic administration of 7,8-DHF leads to behavioural changes [22,23,24] and prolonged central TrkB activation [25,26]. 

Following active lever extinction, rats were reinstated to alcohol in a final 20-min operant session. Thirty minutes prior to testing, rats that received daily 7,8-DHF injections were given a final IP injection of 10 mg/kg 7,8-DHF. Rats that were given a vehicle IP injection over the last 2 weeks were similarly given a vehicle injection 30 min prior to testing. The vanilla essence and stimulus light cues were reintroduced to the chamber and a single drop of ethanol was placed within the receptacle that typically dispensed the alcohol solution in previous phases of the testing protocol. Active presses were recorded in this final session and compared to the extinction values to determine alcohol craving and reinstatement/relapse propensity.

### 2.6. Locomotor Activity

Spontaneous locomotor activity was recorded immediately following the final reinstatement session to examine whether 7,8-DHF treatment had any effect on overall behavioural activity, such as hyperactivity or lethargy, which could impact lever-pressing activity. All rats were tested in locomotor photocells (H:31 × W:43 × L:43cm; MED Associates, St. Albans, VT, USA) over a single 20-min session, directly following the end of the reinstatement session.

### 2.7. Statistical Analysis

Data are presented as the mean ± standard error of the mean (SEM). Statistical analysis was done with Statistical Package for Social Sciences (SPSS, IBM Corp, Armonk, NY, USA). There were initially four groups of 12 rats: male vehicle-treated, male 7,8-DHF-treated, female vehicle-treated, and female 7,8-DHF-treated; final numbers of animals per group are indicated in the figure legends. Data were compared using mixed analysis of variance (ANOVA) with Sex and Treatment (7,8-DHF vs. vehicle) as between-group factors and, where appropriate, Time (FR3) or Session (comparison of extinction vs. reinstatement) as within-group factors. The Greenhouse–Geisser correction was applied where appropriate. Univariate ANOVA was used for between-group comparisons only if the mixed ANOVA showed significant interactions. A *p* value ≤ 0.05 was considered statistically significant.

## 3. Results

**Figure 2:**
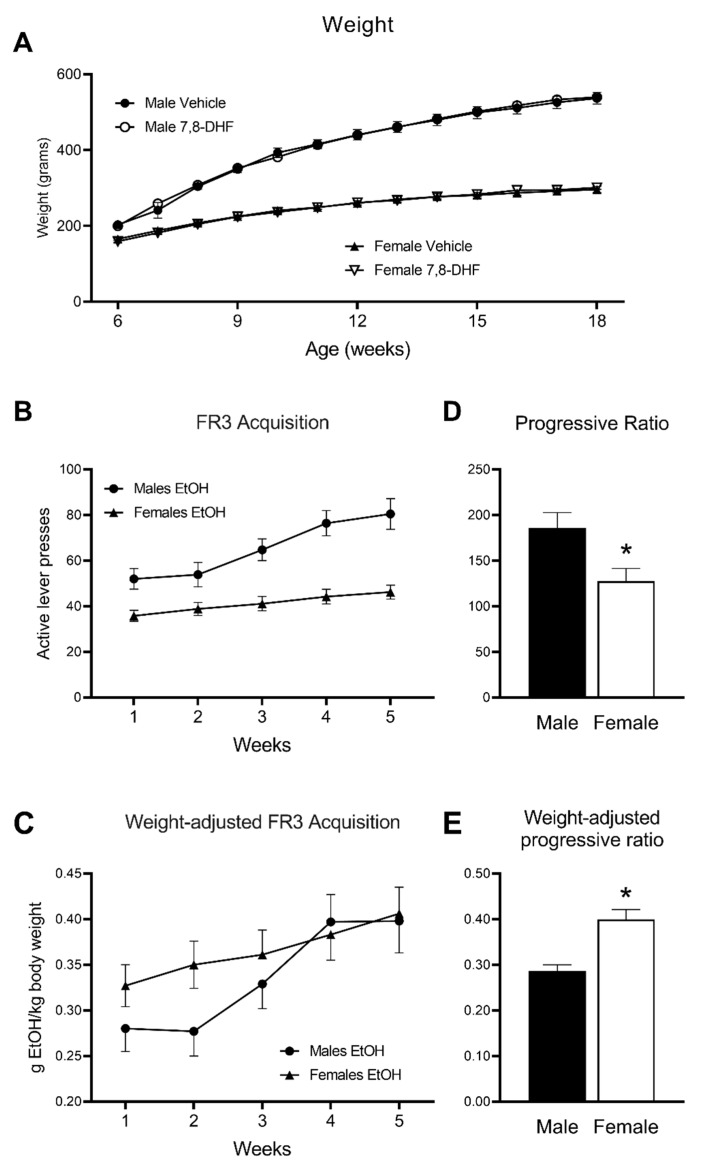
(**A**) Average weight of male and female SD rats across the duration of operant ethanol self-administration. Male rats were significantly heavier than female rats at all time points, but no significant differences were detected between vehicle and 7,8-DHF treatment groups. (**B**) Operant responding at FR3 to a 10% *v*/*v* ethanol solution in SD rats. Acquisition rate was similar across sexes although males had significantly higher numbers of active lever presses. (**C**) Weight-adjusted amount of ethanol ingested at FR3. Acquisition rate was faster in males than in females, but at 5 weeks there were no significant sex differences. (**D**) Breakpoint number of active lever pressing for a 10% *v*/*v* ethanol solution was significantly higher in male compared to female rats. (**E**) Weight-adjusted breakpoint amount of ethanol ingested per body weight was significantly higher in female rats than in males. In all cases, data are mean ± SEM. The number of animals was *n* = 12 in each group in 2A and *n* = 24 in each group elsewhere. * = *p* < 0.05 for difference between males and females.

All 48 rats successfully learned to operate the levers within the chamber during the overnight training and sucrose fade (data not shown). There were no significant weight differences between treatment groups although, as expected, males weighed significantly more than females throughout the experiment (main effect of Sex: F(1,46) = 437.7, *p* < 0.001; Weight change × Sex interaction; F(2.5,114.7) = 19.3, *p* < 0.001; Figure 2A).

Baseline acquisition of lever pressing for a 10% ethanol solution at FR3 was achieved after approximately 25 days of testing. During this acquisition period, operant self-administration, expressed as the number of active lever presses, gradually increased (main effect of Time: F(2.7,124.4) = 41.6, *p* < 0.001) and was consistently higher in males than in females (main effect of Sex, F(1,46) = 19.5, *p* < 0.001; Time × Sex interaction: F(2.7,124.4) = 12.5, *p* < 0.001). At Week 5, males pressed an average of 80.5 ± 6.7, compared to females 46.2 ± 3.1 (F(1,44) = 22.9, *p* < 0.001). Despite marked differences in lever pressing numbers, both male and female rats appeared to reach a stable acquisition within a similar timeframe, with only marginal increases in both groups’ responding between the 4th and 5th week of training (Figure 2B). 

To take into account the significant sex differences in body weight (Figure 2A), data were also analysed as the ratio of the amount of ethanol ingested by body weight (Figure 2C). This amount increased over time (main effect of Time: F(3.1,140.8) = 25.2, *p* < 0.001) and, while there was no main effect of Sex, this number increased more rapidly over time in males than in females (Time × Sex interaction: F(3.1,140.8) = 3.64, *p* = 0.014). At Week 5, there was no sex difference in the amount of ethanol ingested expressed as a ratio of body weight (Figure 2C). Males ingested an average of 0.398 ± 0.035 g ethanol/kg body weight, compared to females 0.406 ± 0.029 g/kg.

Significant differences were observed between male and female rats for progressive ratio (PR). Male rats pressed the active lever significantly more during the 90-min session than female rats (F(1,46) = 7.14, *p* = 0.010; Figure 2D), suggesting a higher breakpoint and persistence to consume alcohol in male rats. However, when accounting for the significant difference in body weight between male and female rats, the amount of ethanol ingested as a ratio of body weight showed a significantly higher breakpoint in females than in males (F(1,46) = 18.4, *p* < 0.001, Figure 2E).

**Figure 3:**
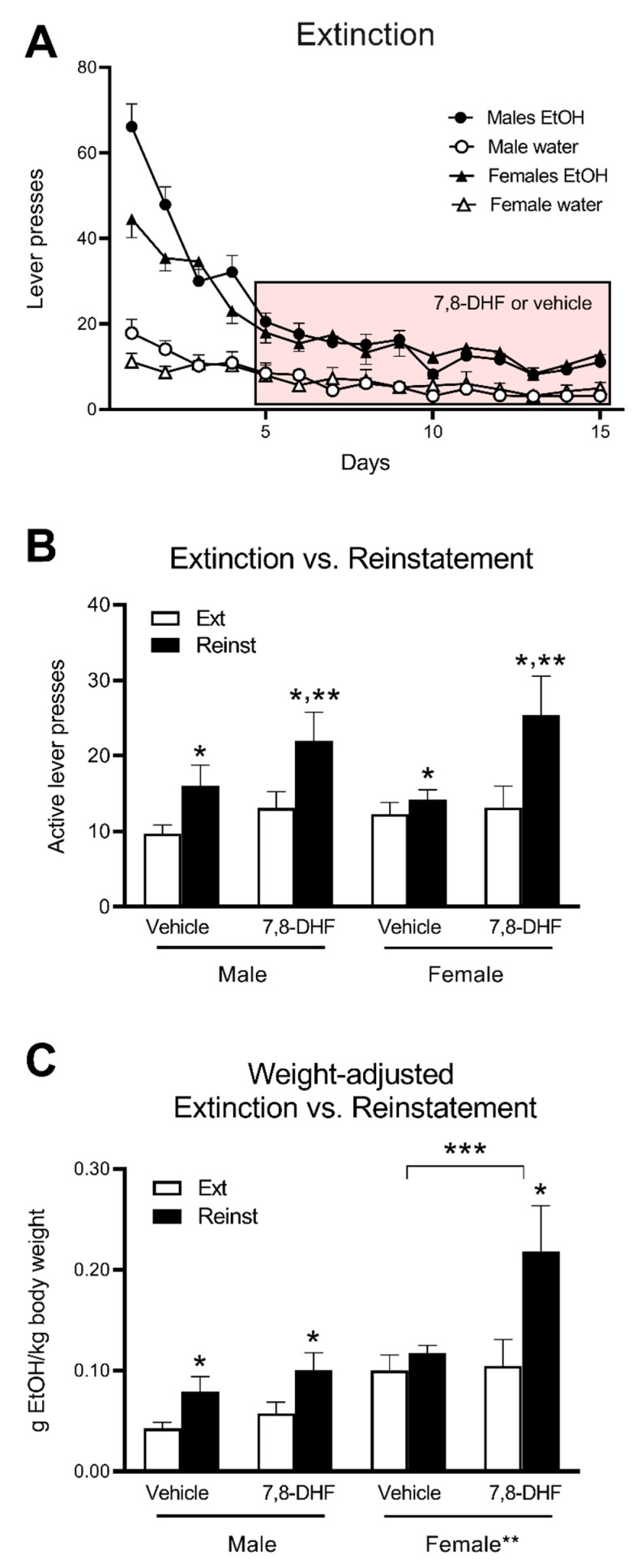
(**A**) Operant responding in male and female SD rats over the 15-day extinction protocol. The red box indicates the injection period of either 5 mg/kg 7,8-DHF or vehicle. (**B**) Average active lever pressing in the last week of extinction (Ext) vs. the reinstatement (Reinst) session. * *p* < 0.05 for difference between number of active lever presses during reinstatement vs. extinction based on the main ANOVA effect of Session. ** *p* < 0.05 for enhanced reinstatement in 7,8-DHF treated compared to vehicle-treated rats based on main effect of Treatment. There were no sex differences. (**C**) Average potential amount of alcohol ingested expressed as ratio of body weight (kg). ** *p* < 0.05 for main ANOVA effect of Sex indicating higher amount of g ethanol/kg in females than in males. * *p* < 0.05 in males for main ANOVA effect of Session with no effect of 7,8-DHF; difference only observed after 7,8-DHF treatment in females. *** *p* < 0.05 for ANOVA Session × Treatment interaction showing higher weight-adjusted g ethanol/kg vs. extinction in female rats following 7,8-DHF than vehicle treatment. All data are mean ± SEM of *n* = 10 for males and *n* = 9 for females.

Following PR and a one-week period of FR3 re-acquisition, rats successfully extinguished lever pressing in response to reward and stimulus cue removal (Figure 3A). Active lever presses markedly fell from the first day of extinction (average 66.1 ± 5.3 in males, 44.5 ± 4.3 in females) to low stable levels during the last five days of the protocol (average 10.5 ± 1.1 in males, 11.9 ± 1.3 in females), although a small, but significant, preference for the previously rewarded lever remained (average previously inactive lever presses 3.5 ± 0.6 in males, 4.7 ± 1.2 in females, main effect for comparison with previously active lever: F(1,44) = 128.7, *p* < 0.001). 

Daily injections of 5 mg/kg 7,8-DHF or vehicle were introduced from Day 5 onwards. There was no significant difference in operant responding during extinction between the drug treatment groups (data not shown).

Of the 48 rats that completed the extinction protocol, 39 reinstated their alcohol drinking behaviour following reintroduction of the stimulus cues. The nine rats that did not respond to the reinstatement session were removed from further analyses (two male vehicle, two male 7,8-DHF, two female vehicle, three female 7,8-DHF) based on activating ≤ 2 rewards (five or fewer presses) over the 20-min period. This cut-off prevented accidental activation of the lever impacting the mean reinstatement value of each group. All groups showed significantly higher numbers of active lever presses during the reinstatement session compared to the extinction session (main effect of Session; F(1,35) = 26.9, *p* < 0.001). Importantly, a significant Treatment x Session interaction was observed (F(1,35) = 5.2, *p* = 0.029), reflective of enhanced reinstatement following the administration of 7,8-DHF compared to vehicle (Figure 3B). However, there were no statistical interactions with Sex (Figure 3B).

To take into account the significant difference in body weight between male and female rats, active lever press data were converted to the potential amount of ethanol ingested and expressed as a ratio of body weight (Figure 3C). Analysis of these data again showed significant reinstatement (main effect of Session: F(1,35) = 29.6, *p* < 0.001) which was significantly greater following 7,8-DHF treatment (Drug × Session interaction: F(1,35) = 6.97, *p* = 0.012). There was also a main effect of Sex (F(1,35) = 13.1, *p* = 0.001) and a Drug x Session x Sex interaction (F(1,35) = 5.48, *p* = 0.025), suggesting differential effects between males and females. Separate analysis of data from males and females revealed that male rats showed significant reinstatement (main effect of Session: F(1,18) = 14.6, *p* = 0.001) but there was no effect of 7,8-DHF treatment (Figure 3C). Female rats also showed significant reinstatement (F(1,17) = 15.6, *p* = 0.001), but this was significantly greater following 7,8-DHF treatment compared to vehicle (Treatment × Session interaction: F(1,17) = 8.46, *p* = 0.010). Post hoc analysis showed significant reinstatement in female rats treated with 7,8-DHF (F(1,8) = 17.2, *p* = 0.003) but not vehicle (Figure 3C). 

Thus, a differential effect of 7,8-DHF on reinstatement between male and female rats was shown, but only following the analysis of reward data as the ratio of the amount of ethanol potentially ingested per body weight (Figure 3B vs. Figure 3C).

**Figure 4:**
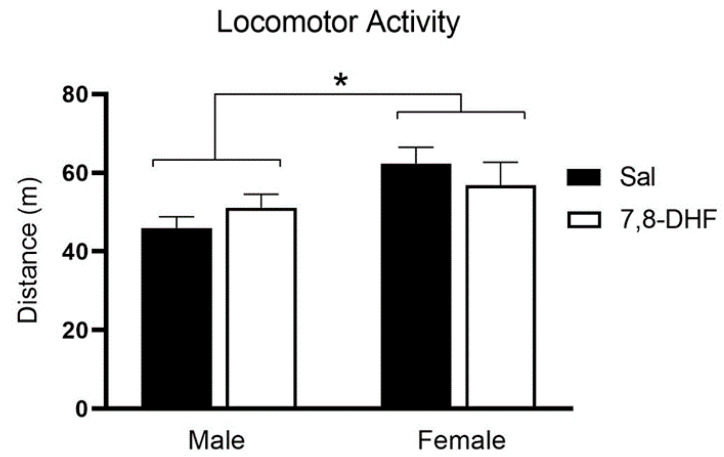
Average spontaneous locomotor activity during a 20-min post-reinstatement session. Rats received either 10 mg/kg 7,8-DHF or sham injection 50 min prior to locomotor testing. Female rats showed higher spontaneous locomotor activity than male rats but there were no differences between 7,8-DHF-treated and vehicle-treated rats. Data are mean ± SEM; * = *p* < 0.05 for difference between males and females.

Spontaneous locomotor activity was not significantly different between control and 7,8-DHF treated rats following reinstatement testing (Figure 4). Female rats had significantly higher locomotion over the 20-min testing session (sex main effect; F(1,35) = 5.3, *p* = 0.027), covering an average distance of 59.6 ± 17.4 m compared to males 48.6 ± 11.1 m.

## 4. Discussion

In line with our previous ethanol self-administration protocol, SD rats successfully learned to respond to stimulus cues and preferentially activate the alcohol-paired lever. Furthermore, lever pressing frequency increased with the enhanced difficulty of obtaining a reward during a progressive ratio protocol, extinguished in response to cue and reward withdrawal, and reinstated with the reintroduction of these stimuli. The absolute number of active lever presses during reinstatement was significantly higher in male and female rats treated with 7,8-DHF compared to vehicle, suggesting a potential role for TrkB activation in alcohol relapse. When expressed as weight-adjusted amount of ethanol ingested, female rats showed significantly higher reinstatement following 7,8-DHF treatment compared to vehicle, whereas this was not observed in male rats. Spontaneous locomotor activity, assessed immediately following the reinstatement session, showed no significant difference between 7,8-DHF and vehicle-injected rats, suggesting any variation in lever pressing between the groups cannot be attributed to non-specific drug-induced hyperactivity.

The data confirm a role of BDNF signalling in the reinstatement of alcohol self-administration after extinction. The previous literature on neurotrophic factors and their role in alcohol addiction highlights an upregulation of BDNF mRNA and protein expression in response to prolonged alcohol intake, followed by a gradual decrease in expression. Previous studies in rats have shown a reduction in BDNF expression typically accompanies increased alcohol craving. This has been demonstrated using BDNF antisense oligonucleotides microinjected into the central amygdala (CeA), which provoked anxiety-like behaviours and increased alcohol preference in a two-bottle choice paradigm [27]. Further evidence of a role for CeA BDNF in alcohol use disorders has been demonstrated by innate differences in BDNF expression between alcohol-preferring (P rats) and non-preferring rats, with P rats having naturally lower BDNF levels within the medial temporal lobe [28]. The same trend in BDNF expression change is seen within the hippocampus, as BDNF levels are enhanced following acute intermittent alcohol exposure but reduced during chronic intake [29]. 

The mesocorticolimbic dopamine system is a central pathway governing learning processes associated with stimulus reward [30,31]. BDNF mediates dopaminergic activity via the trophic modulation of neuron maintenance and differentiation [32]. Therefore, the disruption of BDNF expression and TrkB activation within dopamine-driven reward regions may be critical for AUD development. A study in alcohol naive P rats demonstrated reduced BDNF protein in the NAc [10], while a different study was unable to detect significant levels of BDNF in the ventral striatum, instead reporting on reductions in amygdaloidal BDNF mRNA and protein expression [28]. These findings demonstrate that BDNF may act on VTA-NAc pathways, mediating alcohol addiction and craving propensity. However, because in this study we injected 7,8-DHF systemically, the effects of the drug outside of the dopaminergic pathways cannot be excluded.

Based on previous research, we anticipated that 7,8-DHF-mediated TrkB activation would reduce cue-conditioned alcohol reinstatement. The finding that the TrkB agonist had the opposite effect and promoted reinstatement to alcohol was therefore unexpected. However, recent research has demonstrated BDNF expression recovery following prolonged drug abstinence. BDNF expression within the cortex returns to basal levels during abstinence following exposure to ethanol for <6 weeks in mice [9]. Consequently, the introduction of 7,8-DHF may have enhanced activation of the BDNF receptor above baseline levels and enhanced the addiction phenotype that is previously also described in cocaine studies [33]. If BDNF had normalised in our alcohol-extinguished rats, TrkB activation may have been enhanced and the homeostatic balance between TrkB and P75NTR signalling disrupted, leading to potential addiction/craving reinforcement. This has been demonstrated with other substances of abuse, namely heroin, cocaine, and morphine, where exogenous BDNF within the VTA increased the likelihood of rats becoming drug-dependent [30]. 

Another possibility to explain the apparent discrepancy between enhanced reinstatement in BDNF heterozygous rats in our previous study [14] and the present results is that prolonged treatment with 7,8-DHF resulted in desensitization of the TrkB receptor, effectively inducing a reduced BDNF-TrkB signalling comparable to BDNF heterozygosity. However, previous studies have suggested that 7,8-DHF treatment increases TrkB phosphorylation even after prolonged treatment [25,26]. Future studies will need to confirm if this is the case in animals treated and tested according to the protocol used in the present study.

In our previous study in BDNF HET rats, we found enhanced reinstatement in female but not male rats [14]. Previous studies have shown a close interaction of sex steroid hormones and BDNF signalling [34]. The estrous cycle is known to impact BDNF mRNA expression levels in the hippocampus and frontal cortex of rats, with both reduced and enhanced levels observed during periods of high estradiol circulation [35,36]. The finding that 7,8-DHF enhanced the absolute number of active lever presses similarly in both male and female SD rats was therefore unexpected. However, while BDNF HET rats have significantly reduced BDNF levels in the brain, we previously demonstrated unaltered TrkB phosphorylation in these animals, suggesting possible compensation due to the lifelong deficit in BDNF levels [37]. It is possible that this compensation is different in male and female BDNF HET rats, contributing to sex-specific changes in reinstatement following prolonged BDNF dysregulation in that model. In the present study, 7,8-DHF treatment was continued for a more limited time, possibly preventing global and sex-specific compensatory mechanisms.

Surprisingly, when expressed as the amount of ethanol potentially ingested as a ratio of body weight, a sex-specific effect of 7,8-DHF was found. Compared to vehicle, in female rats 7,8-DHF significantly increased the potential weight-adjusted amount of ethanol per body weight, a treatment effect no longer significant in male rats. Thus, expressing the data this way produced the same sex-specificity as in our previous study [14] although, as discussed above, the direction of the effect of BDNF heterozygosity and 7,8-DHF treatment was unexpectedly similar. Although, analogous to the majority of the literature, we did not express our data adjusted by body weight in our previous study, other reports have shown the importance of this adjustment when evaluating age and sex differences in operant alcohol self-administration [38,39,40,41]. This finding is consistent with the large literature on sex differences in addiction to a range of psychoactive substances [40,42]. The reason for the apparent discrepancy in sex-specificity between the absolute number of lever presses and body weight-adjusted amount of ethanol (potentially) ingested in our study remains unclear, but could be related to the higher level of weight-adjusted responding during the late phase of extinction in female rats compared to male rats (Figure 3C), representing higher activity of the brain circuitry involved in operant responding for alcohol and a resulting greater sensitivity to treatments such as 7,8-DHF. However, in the absence of further studies, this explanation remains speculative. 

There are a number of limitations regarding the study design that should be addressed in future studies. It should be acknowledged that the use of operant self-administration, as opposed to the two-bottle choice paradigm, restricts alcohol access to daily 20-min intervals as opposed to 24-h access. However, the benefit of operant administration is that it permits analysis of motivation-based behaviour, which provides the opportunity to examine potential BDNF-mediated alterations in alcohol craving, where we previously observed a difference in BDNF HETs. Another important limitation to this study is the method of TrkB administration. Although 7,8-DHF is orally bioavailable and capable of rapid Blood–Brain-Barrier diffusion, IP injection would not have resulted in region-specific enhanced TrkB signalling. To address the possible divergent effects of BDNF expression within different neuronal pathways, future studies could target the administration of 7,8-DHF to areas predominantly associated with reward pathways within the brain. This would allow examination of the effect of enhanced TrkB activation in different functionally significant regions such as the cortex, VTA, and NAc. Although this was a behavioural study, future experiments should also incorporate manipulation check to ensure 7,8-DHF was enhancing TrkB phosphorylation, and blood alcohol testing to determine intoxication levels in rats. Finally, given the 2-week abstinence from alcohol over the extinction period, rats’ BDNF levels may have normalised. Therefore, in future it may be beneficial to commence 7,8-DHF treatment during FR3 testing to examine the escalation of habitual alcohol consumption in response to enhanced TrkB activation. This is arguably as important as attempting to prevent alcohol reinstatement, as early intervention is often more efficacious than attempting to undo the persistent neurocellular adaptations leading to prolonged addiction.

## 5. Conclusions

In conclusion, our findings support a role for BDNF in AUD, with activation of the BDNF receptor, TrkB, enhancing relapse/reinstatement. When the number of active lever presses were adjusted for body weight, the effect of 7,8-DHF was specific for female rats. Further work is required to understand the mechanisms involved in the effect of 7,8-DHF, including the regions critical in mediating the involvement of BDNF and TrkB in AUD development and addiction pathophysiology. These studies could lead to targeted neurotrophic interventions to reduce AUD prevalence, particularly in females.
